# Deep learning for synthetic PET imaging: a systematic mapping review of techniques, metrics, and clinical relevance

**DOI:** 10.1186/s41747-025-00651-5

**Published:** 2026-02-09

**Authors:** Maria Vaccaro, Enrico Rosa, Elisa Placidi, Alessia Guarnera, Aurelio Secinaro, Carlo Gandolfo, Maria Carmen Garganese, Antonio Napolitano

**Affiliations:** 1 Medical Physics Unit, Bambino Gesù Children’s Hospital IRCCS, Rome, Italy; 2 UOC Fisica per le Scienze della Vita, Dipartimento di Diagnostica per Immagini e Radioterapia Oncologica, Fondazione Policlinico Universitario A. Gemelli IRCCS, Rome, Italy; 3 eCampus University, Novedrate, Italy; 4 Functional and Interventional Neuroradiology Unit, Bambino Gesù Children’s Hospital IRCCS, Rome, Italy; 5 Advanced Cardiothoracic Imaging Unit, Bambino Gesù Children’s Hospital IRCSS, Rome, Italy; 6 Nuclear Medicine Unit/Imaging Department, Bambino Gesù Children’s Hospital IRCCS, Rome, Italy

**Keywords:** Artificial intelligence, Deep learning, Magnetic resonance imaging, Positron emission tomography, Tomography (x-ray computed)

## Abstract

**Background:**

Synthetic positron emission tomography (PET) imaging, enabled by deep learning, represents a promising approach to minimize radiation exposure while preserving diagnostic accuracy. However, variability in methodologies, performance metrics, and clinical applications needs to be assessed. This systematic mapping review examines the current state of research in synthetic PET generation, analyzing their methodological frameworks and evaluating the clinical relevance.

**Materials and methods:**

A systematic search in Scopus, PubMed, and Google Scholar (2019–2024) identified peer-reviewed studies on deep learning-based synthetic PET. Review articles, conference abstracts, and inaccessible full texts were excluded. Data extraction covered study characteristics, imaging modalities, architectures, and evaluation metrics. Due to study heterogeneity, the risk of bias was not formally assessed. Results were synthesized through descriptive and quantitative analysis.

**Results:**

Of the initial 116 studies retrieved, 34 were included, 25 of them (73.5%) on brain/neuro using magnetic resonance imaging, computed tomography, or low-dose PET data to generate full-dose or tracer-specific PET. Common architectures included convolutional neural networks, generative adversarial networks, and U-Nets. Peak signal-to-noise ratio (PSNR) ranged 22.69–56.87 dB, structural similarity index measure (SSIM) 0.38–1.00 and mean absolute error (MAE) 1.37–72.00%. Whole-body applications were less frequent (9/34, 26.5%) but showed improvements in oncologic imaging, in particular for tumor detection and image quality. Despite promising advancements, challenges remain, including limited data availability, variability in tracer uptake, and the lack of standardized evaluation metrics. The absence of large/multicenter datasets limits the generalizability of findings.

**Conclusions:**

This review highlights promising advancements in synthetic PET imaging using deep learning, with several studies demonstrating the potential for high-quality image generation and substantially reduced radiation exposure. These developments are particularly significant in pediatric populations, where minimizing radiation dose is crucial to ensure patient safety and long-term health. Nonetheless, methodological variability and limited clinical validation continue to pose substantial challenges. Future research should prioritize the development of standardized evaluation protocols, the use of larger and more diverse datasets—including pediatric cohorts—and comprehensive real-world clinical validation to support the safe and effective translation of synthetic PET techniques into clinical practice.

**Relevance statement:**

Deep learning-based synthetic PET imaging enhances diagnostics while reducing radiation, but requires methodological standardization and clinical validation for broader adoption.

**Key Points:**

- Deep learning can create full-dose PET images with less radiation exposure.
- Neurological applications dominate synthetic PET research, maintaining essential diagnostic detail.
- Challenges include limited datasets and variability in tracer uptake, necessitating further advancements.

**Graphical Abstract:**

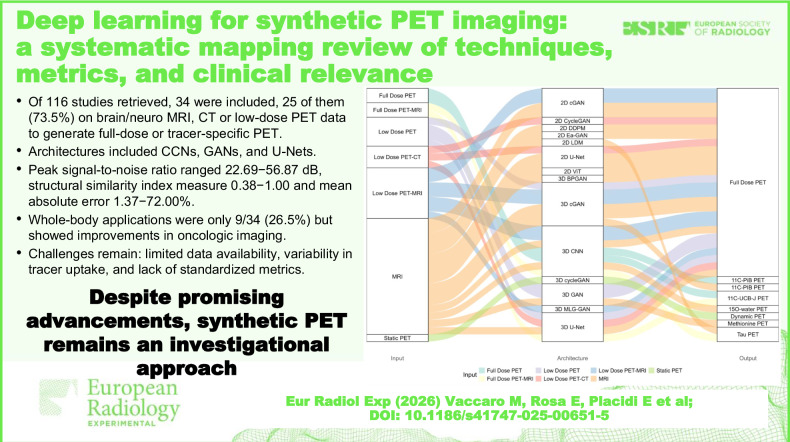

## Background

Positron emission tomography (PET) is a key imaging tool in neurology and oncology, providing metabolic and functional information for disease diagnosis and treatment monitoring [1]. However, its widespread clinical use is limited by high costs, long acquisition times, and radiation exposure from radiotracers. These challenges are particularly relevant in vulnerable populations such as children and pregnant women, or patients requiring serial scans [2].

Recent advances in deep learning (DL) have enabled the generation of synthetic PET images from alternative imaging modalities, such as magnetic resonance imaging (MRI), low-dose PET, or computed tomography (CT), offering a potential solution to reduce radiation exposure while preserving diagnostic accuracy [3]. Various DL architectures, including convolutional neural networks (CNNs), generative adversarial networks (GANs), and U-Net models, have been explored for this purpose [4–7]. These approaches aim to produce synthetic PET images that closely resemble ground-truth PET scans, ensuring high image quality as measured by metrics such as “peak signal-to-noise ratio” (PSNR), “structural similarity index” (SSIM), “mean absolute error” (MAE), and “relative root mean square error” (rRMSE). Despite promising results, methodological variability, inconsistent evaluation metrics, and limited clinical validation hinder the widespread adoption of synthetic PET imaging.

In the initial stages of medical image synthesis, architectures such as autoencoders and fully convolutional networks were commonly employed, as demonstrated by Nie et al in 2016 [8] and Xiang et al in 2017 [9]. These early models focused on generating synthetic images but faced limitations in their ability to capture complex anatomical and functional relationships. Subsequently, more sophisticated architectures emerged, with U-Net and GANs becoming prominent [10], due to their effectiveness in preserving spatial hierarchies within image data, useful for synthesizing high-quality PET images from input modalities such as MRI, and their capacity to capture the intricate relationships between structural and functional data. For instance, the GLA-GAN model uses global and local modules to enhance synthesis quality, addressing anatomical details at multiple scales [11, 12]. This architectural shift marked a significant improvement in synthetic PET image quality, particularly in the context of full-dose PET synthesis from low-dose PET inputs.

In recent years, state-of-the-art models, such as Vision-Transformers and Diffusion-based architectures, have marked a significant advancement in the next wave of image generative networks. These models represent a turning point from traditional designs as described by Khader et al [13] and Kazerouni et al [14], who highlighted their efficacy in handling complex imaging data, including PET. Vision-Transformer-based methods, in particular, have been demonstrated to incorporate global attention mechanisms, establishing pixel-wise relationships between MRI and PET images [15]. Similarly, diffusion models have shown promising results in PET synthesis, especially in scenarios where high-quality image generation is mandatory [16]. These advanced models exemplify the cutting-edge techniques currently used in medical image synthesis, reflecting the evolution of the field from basic autoencoder-based designs to more sophisticated methods.

Medical image synthesis, particularly synthetic PET, has become a robust tool for avoiding repeated scans with high radiation exposure, particularly for pediatric patients who face increased risks of developing brain cancers and leukemia [17]. The application of synthetic PET is highly relevant in scenarios such as diagnosing degenerative disorders, including Alzheimer’s disease, where the cerebral distribution of fluorodeoxyglucose (FDG) in PET scans serves as a crucial differentiating factor [18].

Furthermore, synthetic PET enhances the diagnostic accuracy of cerebrovascular diseases by utilizing MRI-derived cerebral blood flow maps [19]. It also plays a significant role in developing PET reconstruction algorithms, where synthetic data is used for training and evaluation [20].

Although several reviews have addressed DL approaches for PET image synthesis, to date, no systematic review has comprehensively mapped and analyzed the newest existing literature to assess the performance, clinical feasibility, and diagnostic accuracy of DL-based synthetic PET generation. This review aims to fill this gap by systematically evaluating existing studies, comparing synthetic PET generation from different input images (*e.g*., MRI-to-PET and CT-to-PET), and identifying the most effective computational approaches. Furthermore, we argue that a formal quality assessment of the papers is a key factor to avoid any scattered results, thus showing a clearer pathway toward optimal solutions. To this end, we provide a structured overview of the field, highlight key limitations, and outline directions for future research and clinical translation while keeping the readers informed on the pitfalls and the quality of the state of the art in PET data generation.

## Materials and methods

### Literature search

A systematic literature search was conducted using Scopus and PubMed, covering studies published between January 2019 and September 2024. To ensure completeness, Google Scholar was also consulted. The search focused on studies investigating synthetic PET imaging using DL, with keywords such as “synthetic PET” AND “deep learning” applied to titles, abstracts, and keywords. Only English-language peer-reviewed articles were considered. The term ‘PET’ is used consistently to refer to the synthesized PET image component only, rather than the full hybrid imaging system (*e.g*., PET/CT or PET/MRI). This reflects the focus of the included studies, which aim to generate synthetic PET images irrespective of the original modality used for acquisition.

The study selection process was conducted independently by two reviewers, who screened studies in two stages: (1) title and abstract screening to exclude irrelevant studies, and (2) full-text review to assess eligibility. Disagreements were resolved through discussion or, if needed, by consulting a third reviewer. Additionally, backward and forward citation searches were performed to identify additional relevant studies, but they did not result in the inclusion of any additional studies.

### Eligibility criteria

Studies were included if they: (1) investigated synthetic PET generation using DL models; (2) reported quantitative performance metrics such as PSNR, SSIM, MAE, or rRMSE; (3) used medical imaging modalities (MRI, low-dose PET, or CT) as input; (4) were peer-reviewed and had full-text access. Studies were excluded if they: (1) were review articles, conference abstracts, book chapters, or editorials; (2) did not report quantitative performance evaluations of synthetic PET models; (3) focused on other synthetic imaging modalities unrelated to PET.

### Data extraction and analysis

To assess the methodological rigor of the included studies, we employed a checklist inspired by METRICS principles commonly used in AI-centered radiology research [21]. The checklist consists of 11 extracted items covering clinical context, data handling, model development, assessment of DL pipelines, validation, evaluation metrics, reproducibility, and clinical relevance. Each study was evaluated independently across these criteria and assigned a score: 1 for fully satisfied, 0.5 for partially fulfilled, and 0 for unmet. Total scores thus ranged from 0 to 11. Particular attention was paid to external validation and the availability of open-source code or datasets.

For each included study, key details were extracted and systematically organized to facilitate comparison. These details included: input and output imaging modalities (*e.g*., MRI to PET, low-dose PET to full-dose PET), DL architectures (*e.g*., CNNs, GANs, U-Nets, Transformers, Diffusion models), clinical applications (*e.g*., neurology, oncology, whole-body imaging), evaluation metrics (*e.g*., PSNR, SSIM, MAE, rRMSE), publication year and references.

A Kruskal-Wallis test was conducted to compare PSNR values between studies that included external validation and those that did not.

Table 1 provides an overview of key terms, architectures, and evaluation metrics relevant to synthetic PET image generation.

**Table 1:** Glossary of key concepts, architectures, and evaluation metrics in synthetic PET imaging

Term	Definition/description	Clinical relevance/usage
PET	Positron Emission Tomography. Imaging technique providing metabolic/functional information via radiotracer uptake.	Widely used in oncology and neurology for diagnosis and treatment monitoring.
Synthetic PET	Artificially generated PET images from other imaging modalities using deep learning techniques.	Reduces radiation exposure and scan time; useful in vulnerable populations.
MRI-to-PET/CT-to-PET	Generation of synthetic PET from anatomical images, such as MRI or CT.	Radiation-free or reduced-radiation alternative to PET acquisition.
Low-dose PET	PET imaging acquired with reduced radiotracer dose.	Minimizes radiation; synthetic methods aim to recover full-dose quality.
CNN	Deep learning model effective for spatial feature extraction and noise reduction.	Enhances image quality in low-dose-to-full-dose PET synthesis.
U-Net	Encoder-decoder CNN with skip connections, preserving fine-grained image details.	Common in medical image synthesis for retaining anatomical structures.
GAN	Generative adversarial network—Comprises generator and discriminator networks; generates realistic images.	Used in PET synthesis to improve visual similarity and texture realism.
Vision transformer (ViT)	Attention-based deep model capturing long-range dependencies in image data.	Promising in MRI-to-PET synthesis with global context modeling.
Diffusion model	Probabilistic model iteratively refining noise to generate images.	High fidelity and detail preservation in synthetic PET.
PSNR	Peak signal-to-noise ratio—higher values = lower noise.	Measures image clarity; > 40 dB = high-quality in medical imaging.
SSIM	Structural similarity index—compares luminance, contrast, structure (0 to 1).	Closer to 1 = better structural match with reference image.
MAE	Mean absolute error—average pixel-wise error.	Evaluates intensity accuracy; lower = better.
RMSE/rRMSE	Root Mean Square Error/relative RMSE—global/normalized error measures.	Assesses pixel intensity precision.
Dice score	Measures overlap of predicted and reference regions (0 to 1).	Used in segmentation (*e.g*., tumor delineation).
SUV bias	Bias in standardized uptake values—quantifies radiotracer uptake error.	Important for metabolic accuracy in oncology.

*MRI* Magnetic resonance imaging

### Data synthesis and presentation

Both qualitative and quantitative syntheses were conducted to identify trends and assess model performance. Key quantitative performance metrics were compared across different DL architectures and imaging modalities. A subgroup analysis explored differences in input modalities (MRI, low-dose PET, CT) and their impact on synthetic PET generation, model performance in neurological *versus* whole-body PET applications, and variability in reported performance metrics across studies.

Findings were summarized using descriptive statistics and visually represented through bar plots, alluvial diagrams, and comparative tables.

## Results

### Overview of the included literature

The search initially retrieved 116 articles. After removing 38 records that were deemed ineligible (21 conference papers, 10 conference reviews, 6 review articles, and 1 book chapter), 78 potentially relevant articles remained. These were further screened based on full-text availability and relevance to the study, resulting in the exclusion of an additional 44 articles. Ultimately, 34 peer-reviewed articles met the inclusion criteria and were selected for analysis.

About the listed studies, 25/34 of them (73.5%) focus on brain-specific applications, and 9/34 of them (26.5%) specifically on whole-body imaging. The key data regarding the input modality, output modality, DL architecture, clinical focus/district and references are synthetized in Table 2.

**Table 2:** Evaluation metrics for different synthetic PET generation approaches

Authors [reference number]	PSNR (dB)	SSIM	MAE (%)	NMSE (%)	CNR (%)	rRMSE	PCC	SUV	FID	MAPE (%)	MRAE	Accuracy (%)	Sensitivity (%)	Specificity (%)	AUC (%)	MMD
Zhou et al [36]	27.04	0.80	34.27													
Sun et al [54]	32.07	0.99		0.08	0.67											
Xue et al [24]	41.82	1.00				0.12										
Sanaat et al [49]	29.00	0.84				0.40		1.12								
Haggstrom et al [43]	34.69	0.98				0.60										
Wang et al [42]	55.60	0.97				0.16										
Kaplan and Zhou [4]	30.56	0.94				0.27										
Wang et al [47]	25.19	0.98														
Jang et al [33]																
Xie et al [16]	30.92	0.93		0.003												
Zhang et al [52]	27.53	0.93				0.04	0.99									
Shin et al [50]	47.52	0.38				2.38										
Sikka et al [11]	29.33	0.97	1.37													
Wei et al [48]	30.04															
Emami et al [44]	28.45	0.87	46.26													
Hu et al [12]	27.88	0.89	25.34						29.17							
Lin et al [53]	29.42	0.82														
Lee et al [40]		0.90					0.62			0.10						
Wang et al [57]	38.37	0.92	2.14													
Wang et al [38]		0.91				0.16	0.97									
Rajagopal et al [20]		0.94	6.60								0.37					
Chen et al [34]												80.00	78.00	74.00		
Zhang et al [3]												62.45			76.10	
Kim et al [23]	56.87								10.43							
Gao et al [55]	29.10	0.92													92.40	0.08
Miao et al [35]		0.70		0.15												
Abazari et al [37]	30.82	0.82														
Islam and Zhang [51]	32.83	0.77										71.45				
Lei et al [25]	30.40		72.00													
Vega et al [22]	22.69	0.93					0.96									
Hussein et al [19]	38.80	0.92				0.04										
Zhang et al [15]	26.92	0.73	3.18													
Takita et al [56]															81.00	
Ouyang et al [58]	27.83	0.88				0.15										

This table summarizes the performance of various synthetic PET models using key quantitative metrics. Each row represents a study, linking the employed architecture to its performance on specific metrics

*AUC* Area under the receiving operator characteristic curve, *CNR* Contrast-to-noise ratio, *FID* Fréchet inception distance, *MMD* Maximum mean discrepancy, *MAE* Mean absolute error, *MAPE* Mean absolute percentage error, *MRAE* Mean relative absolute error, NMSE Normalized mean square error, *PCC* Pearson correlation coefficient, *PSNR* Peak signal-to-noise ratio, *rRMSE* Relative root mean square error, *SUV* Standardized uptake value, *SSIM* Structural similarity index

**Fig. 1:**
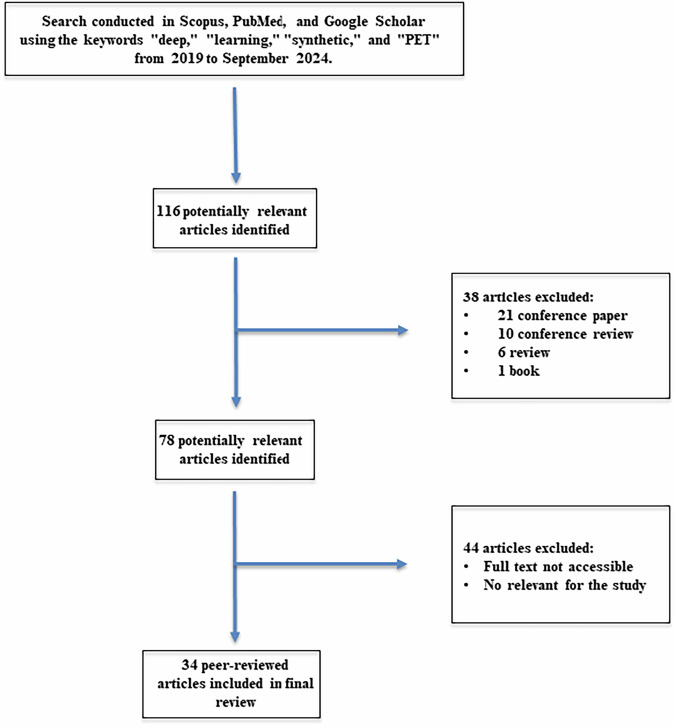
Flowchart of the literature analysis, including the keywords employed for the search, as well as the inclusion and exclusion criteria applied to select the articles

The study selection process is visually represented in the flowchart of Fig. 1.

A detailed scoring table for all 34 studies is provided in Table 3.

**Table 3:** Methodological quality assessment of the 34 studies included in the systematic mapping review

Authors [reference number]	Clinical background	Data transparency	Data split	Model architecture	External validation	Evaluation metrics	Comparison with baseline	Robustness assessment of end-to-end deep learning pipelines	Code availability	Clinical relevance	Discussion of limitations	Total score
Haggstrom et al [43]	1	1	0.5	1	0	1	0	0.5	0	0	0.5	5.5
Kaplan and Zhu [4]	1	0.5	0	1	0	1	0	0	0	0	0.5	4
Wang et al [47]	1	1	1	1	1	1	0	1	0	0	1	8
Chen et al [34]	1	1	1	1	0	1	0	0.5	0	0	1	6.5
Wei et al [48]	1	0.5	0.5	1	0	1	0	0.5	0	0	0	4.5
Sanaat et al [49]	1	1	0.5	1	0	1	0	0.5	0	0	0.5	5.5
Shin et al [50]	1	1	0.5	1	0	1	0	0.5	0	0	0.5	5.5
Emami et al [44]	1	1	1	1	0	1	1	0.5	1	0	0	7.5
Islam and Zhang [51]	1	0.5	0	1	0	1	0	0.5	0	0	0.5	4.5
Xue et al [24]	1	1	1	1	0	1	1	0.5	0	0	0	6.5
Wang et al [38]	1	1	1	1	1	1	0	1	0	0	0	7
Zhang et al [52]	1	0.5	0.5	1	0	1	0	0.5	0	0	0	4.5
Sikka et al [11]	1	1	1	1	0	1	0	1	0	0	0	6
Hu et al [12]	1	1	1	1	0	1	0	1	0	0	1	7
Lin et al [53]	1	1	1	1	0	1	0	0.5	0	0	0	5.5
Wang et al [42]	1	1	1	1	1	1	0	1	0	0	1	8
Zhou et al [36]	1	1	1	1	1	1	0	1	0	0	0	7
Sun et al [54]	1	1	1	1	0	1	0	0.5	0	0	0	5.5
Abazari et al [37]	1	1	1	1	0	1	1	0.5	0	0	0	6.5
Zhang et al [15]	1	1	1	1	0	1	0	0.5	1	0	0	6.5
Jang et al [33]	1	1	1	1	0	1	0	0.5	0	0	1	6.5
Xie et al [16]	1	1	1	1	0	1	0	0.5	0	0	1	6.5
Rajagopal et al [20]	1	1	0.5	1	0	1	0	0.5	0	0	0.5	5.5
Zhang et al [3]	1	1	1	1	1	1	0	0.5	0	0	1	7.5
Kim et al [23]	1	1	1	1	0	1	0	1	0	0	1	7
Gao et al [55]	1	1	1	1	0	1	0	0.5	0	0	0	5.5
Miao et al [35]	1	1	1	1	0	1	0	0.5	0	0	0	5.5
Lei et al [25]	1	1	1	1	0	1	0	1	0	0	0	6
Takita et al [56]	1	1	1	1	1	1	0	1	0	0	1	8
Lee et al [40]	1	1	1	1	1	1	0	0.5	0	0	1	7.5
Wang et al [57]	1	1	1	1	0	1	0	1	1	0	0	7
Vega et al [22]	1	1	1	1	0	1	0	1	1	0	1	78
Hussein et al [19]	1	1	1	1	1	1	0	0.5	0	0	1	7.5
Ouyang et al [58]	1	1	1	1	1	1	1	1	0	1	1	10

The METRICS checklist [59] was used. Each study was evaluated across 11 predefined criteria related to clinical context, data handling, model development, assessment of deep learning pipelines, validation, evaluation, reproducibility, and clinical integration. Scores range from 0 (criterion not satisfied) to 1 (fully satisfied), with 0.5 indicating partial fulfillment. The total score reflects the overall methodological rigor of each study

### Methodological quality assessment

Among the 34 studies included, all provided a clinical background and described the model architecture. Data transparency was reported in 32 studies (94.1%), while data splitting strategies were reported in 29 studies (85.3%). External validation was performed in 10 studies (29.4%). Regarding evaluation metrics, 32 studies (94.1%) reported them clearly. Robustness assessment of end-to-end DL pipelines was fully evaluated in 10 studies (29.4%). Only 6 studies (17.6%) made their code publicly available. Clinical relevance metrics, such as diagnostic accuracy or expert evaluation, were included in 6 studies (17.6%). Discussion of limitations was reported in 9 studies (26.5%). Total METRICS scores ranged from 4 to 10, with a median score of 6.5. Additionally, of the 34 included studies, 10 (29.4%) performed some form of external validation, while only 6 included clinical evaluation metrics such as diagnostic task performance or reader feedback.

### Input modalities and architectures

The selected publications include a wide range of input modalities, such as low-dose ₁₈F-FDG PET (Low-dose PET), MRI, and CT or a combination of these, to generate full-dose ₁₈F-FDG PET (Full-dose PET) or other specific PET modalities. Various DL architectures, including cGANs, CycleGANs, U-Nets, and CNNs, and more recent advancements like vision transformers and denoising diffusion probabilistic models, have been utilized to enhance image synthesis performance, each demonstrating unique capabilities depending on the complexity of the input and the desired output.

### Temporal trends and application fields

**Fig. 2:**
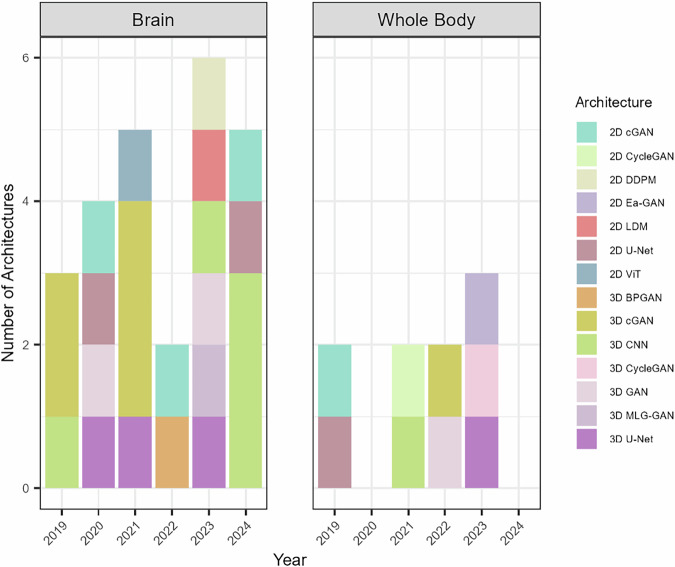
Chronological overview of deep learning-based networks used for PET image synthesis as reported in the literature. The panel on the left shows the number of articles and the types of architectures employed for synthetic PET in the brain region, while the panel on the right displays the corresponding information for whole-body synthetic PET. 2D, Two-dimensional; 3D, Three-dimensional; BPGAN, Backpropagation generative adversarial network; cGAN, Conditional generative adversarial network; CNN, Convolutional neural network; DDPM, Denoising diffusion probabilistic model; Ea-GAN, Edge-aware generative adversarial network; GAN, Generative adversarial network; LDM, Latent diffusion model; MLG-GAN, Multi-level generative adversarial network; ViT, Vision transformer

To visualize the progression of synthetic PET research over time, an analysis was conducted focusing on the use of different DL architectures for brain-specific and whole-body applications shown in Fig. 2. The resulting histogram highlights the distribution of studies from 2019 to 2024, revealing trends in increasing the adoption of such architectures for synthetic PET data creation.

The panel on the left shows the number of articles and the types of architectures employed for synthetic PET in the brain region, while the panel on the right displays the corresponding information for whole-body synthetic PET.

### Input–architecture–output relationships

**Fig. 3:**
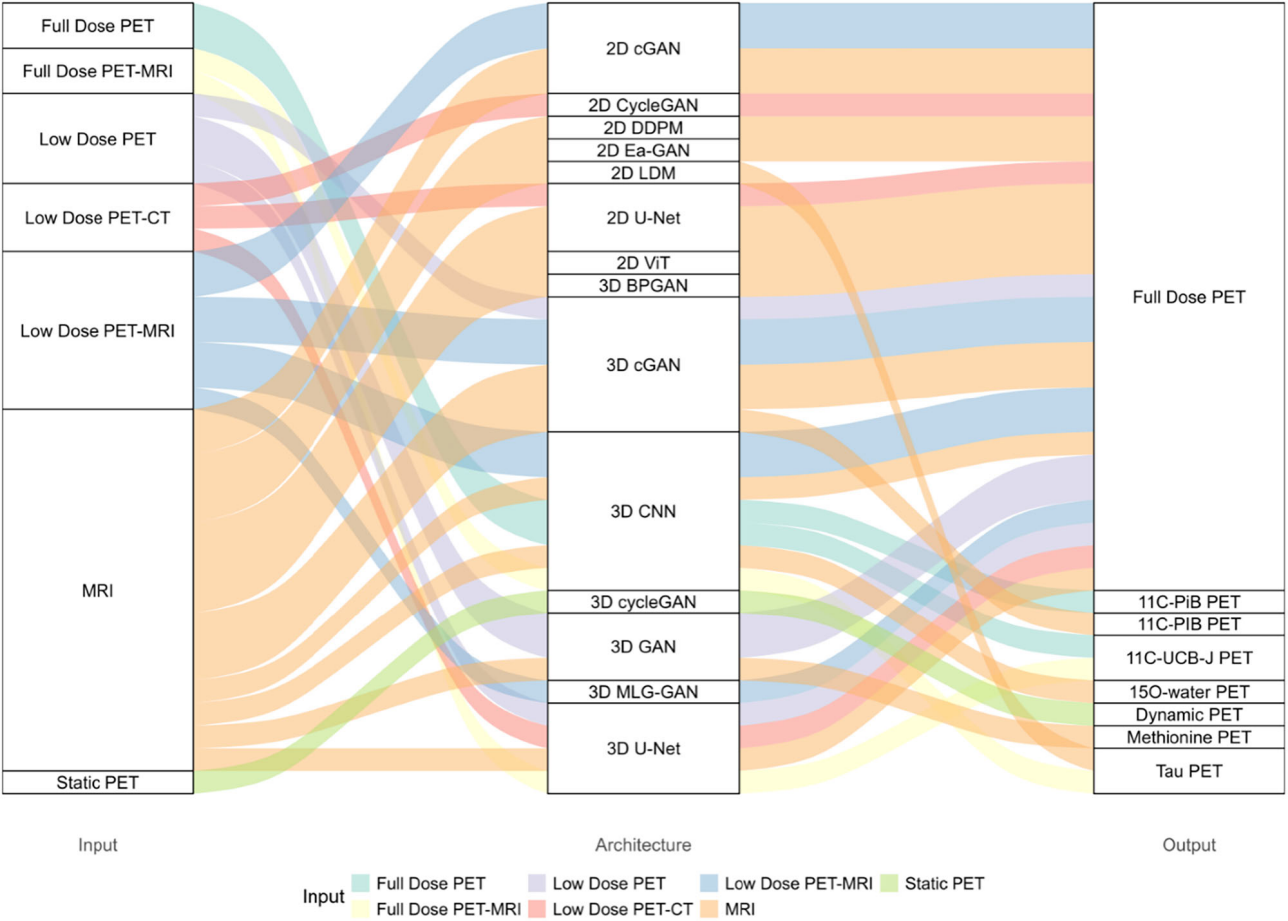
Alluvial plot depicting the relationships between input modalities, network architectures, and output types for PET data synthesis. 2D, Two-dimensional; 3D, Three-dimensional; BPGAN, Backpropagation generative adversarial network; cGAN, Conditional generative adversarial network; CNN, Convolutional neural network; PET, Positron emission tomography; MLG-GAN, Multi-level generative adversarial network; MRI, Magnetic resonance imaging; CT, Computed tomography; cGAN, Conditional generative adversarial network; DDPM, Denoising diffusion probabilistic model; Ea-GAN, Edge-aware generative adversarial network; GAN, Generative adversarial network; LDM, Latent diffusion model; PET, Positron emission tomography; PiB, Pittsburgh compound B; ViT, Vision transformer

The alluvial plot presented in Fig. 3 illustrates the relationships between different input modalities, architectures, and output types in synthetic PET image generation. The plot highlights how various input data are processed through DL architectures, producing different PET outputs. The flow of connections between these components provides a clear visualization of how different architectures are used for specific types of input and output. This plot serves as a visual summary of the diversity of approaches adopted in synthetic PET research, underscoring the field’s ongoing development and innovation in DL methodologies for medical imaging.

**Fig. 4:**
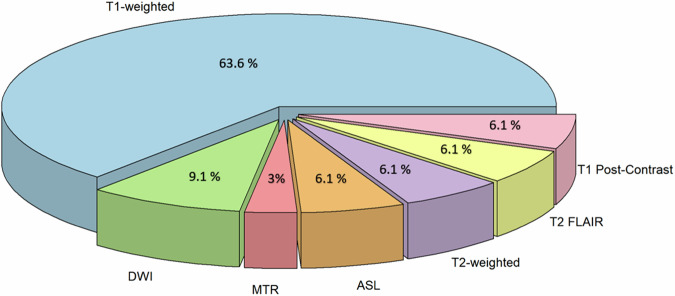
Distribution of the MRI input modalities/sequences used in synthetic PET studies. ASL, Arterial spin labeling; DWI, Diffusion-weighted imaging; FLAIR, Fluid-attenuated inversion recovery; MTR, Magnetization transfer ratio

As evidence of Fig. 3, MRI stands out as the predominant input modality, underscoring the value of exploring the distribution of MRI input types employed in synthetic PET studies. This distribution is shown in the pie chart presented in Fig. 4.

### Quantitative performance metrics

Following the exploration of input modalities, architectures, and output types, we have structured a detailed table (Table 2) that compiles the evaluation metrics for each synthetic PET generation approach, as reported in the reviewed articles. This table provides a breakdown of the performance of different DL models based on key quantitative metrics.

From the table data, the metrics Peak Signal-to-Noise Ratio (PSNR), Structural Similarity Index (SSIM), Mean Absolute Error (MAE), and relative Root Mean Square Error (rRMSE) were selected for further analysis as they appeared most frequently in multiple articles. PSNR ranges from 22.69 dB (Vega et al [22]) to a maximum value of 56.87 dB in the case of Kim et al [23]. The metric SSIM ranges from 0.38 to 1.00, the maximum found by Xue et al [24]. MAE exhibits a range from 1.37% (Sikka et al [11]) to 72% [25]. The values of rRMSE range between 0.04 (Zhang et al, Hussein et al [15, 19]) and 0.27 [4].

To provide a more comprehensive analysis, we calculated the mean values of the metrics considered in our analysis for each input modality used in synthetic PET image generation. These average values highlight the overall performance of neural networks based on the type of input data. For instance, when combining PET with another imaging modality, the mean values are: 35.00 dB for PSNR, 0.89 for SSIM, 34.27% for MAE, and 0.28 for rRMSE. Using MRI alone as the input modality yielded a slightly lower PSNR of 31.28 dB, an SSIM of 0.86, an MAE of 22.41%, and an rRMSE of 0.65. In contrast, PET data alone exhibited a higher PSNR of 36.89 dB and an MAE of 34.27%, though the SSIM was 0.77, and the rRMSE value was not determined.

The analysis did not reveal any statistically significant differences (H = 0.29, *p* = 0.593), indicating that the presence of external validation was not associated with improved PSNR performance in the available data. The test was performed exclusively on PSNR values, as this was the most consistently reported metric across studies. Other evaluation metrics, such as SSIM, MAE, normalized mean square error, or SUV, were reported too infrequently or in heterogeneous formats, preventing a reliable statistical comparison across groups.

## Discussion

This review aimed to systematically analyze and compare DL-based synthetic PET imaging techniques, focusing on their network architectures, performance metrics, and clinical applications. Our findings highlight the rapid advancement of synthetic PET imaging, with significant improvements in image synthesis quality and diagnostic potential. However, challenges such as data scarcity, methodological variability, and generalizability remain key obstacles to widespread clinical adoption.

The data illustrate the evolution of synthetic PET methodologies, from early neural network architectures to advanced techniques, that have significantly improved image quality and diagnostic accuracy. These advancements have enabled precise clinical assessments in fields such as oncology and neurology, addressing diverse medical needs with greater efficacy.

As DL continues to advance, further refinement of these methods is expected, driving the broader adoption of synthetic PET imaging in clinical practice and broadening its applicability across a growing spectrum of medical applications.

Evaluating a neural network designed for synthetic PET image generation requires assessing multiple facets, from image fidelity to its clinical applicability. A high-performing network must accurately replicate the fine structural and metabolic details necessary for making a proper diagnosis, while maintaining robust performance across diverse patient populations and scanning protocols. This evaluation goes beyond basic image quality, extending into more nuanced criteria to ensure the neural network is both effective and clinically viable. One of the most important factors in assessing a neural network is its ability to reduce noise while preserving the anatomical features of the image. CNNs, for instance, are particularly effective at enhancing image quality by suppressing noise without blurring key structural elements. This noise reduction is crucial for synthetic PET images generated from low-dose scans, where artefacts can otherwise distort the data and undermine diagnostic utility [26]. Another key aspect is the network’s ability to generalize well across various datasets in order to ensure consistent performance, as overfitting to a single dataset can reduce the model’s applicability to broader clinical environments. Testing across different scanners, imaging protocols, and patient populations is therefore critical to ensure the robustness of the synthetic PET images.

To evaluate neural networks used for synthetic PET image generation, several quantitative metrics are typically employed to assess both image quality and clinical utility. The PSNR quantifies image clarity by comparing signal strength to noise levels. Higher PSNR values indicate superior image quality, making it a reliable measure for comparing synthetic images to their ground-truth counterparts. In research, PSNR values exceeding 40 are often considered indicative of high-quality images in medical imaging applications [27].

Another important metric is the SSIM, which assesses how closely the synthetic image matches the real image in terms of luminance, contrast, and structure. SSIM values range from 0 to 1, with values closer to 1 indicating better image quality and preservation of structural integrity [28].

The MAE quantifies the average pixel-level difference between the synthetic and real PET images, providing insight into the overall accuracy of the model. Lower MAE values indicate better performance, as they reflect a closer match between the synthetic image and the ground-truth data. This metric is particularly useful for assessing how well the model captures small details, which is crucial in medical imaging, where subtle differences can impact clinical decisions [29].

For instance, in synthetic PET image evaluations, MAE is often used alongside other metrics like PSNR and SSIM to ensure that the overall error is minimized and the visual and structural integrity of the image is maintained.

The RMSE is the metric used to evaluate the overall error between predicted and actual images in synthetic PET image generation. It is particularly useful for quantifying the differences in pixel intensity between synthetic images and ground-truth images. A lower RMSE value reflects a higher degree of accuracy in image synthesis, making it an essential measure for ensuring that synthetic PET images closely replicate the original PET scans. Similar to MAE, RMSE is also used with other metrics like SSIM and PSNR in evaluating cross-modality image synthesis, where the task is to maintain precision in intensity and image quality [30].

For segmentation tasks in synthetic PET imaging, the Dice score is commonly used to evaluate how well the predicted regions of interest, such as tumors or lesions, overlap with the ground-truth anatomical structures. This metric is particularly useful in assessing the network’s ability to detect and delineate abnormalities accurately. The Dice score measures the similarity between the predicted segmentation and the true regions, with values closer to 1 indicating a high degree of overlap and accurate segmentation [31, 32].

Lastly, the Standardized Uptake Value Bias (SUV Bias) is useful in evaluating radiotracer uptake in synthetic PET images, especially in oncology. This metric ensures that synthetic images accurately reflect metabolic activity [27].

Incorporating these metrics ensures that the neural networks produce visually accurate synthetic PET images and maintain the clinical relevance required for real-world medical applications.

The results of the METRICS evaluation underscore several key gaps in the current body of work on synthetic PET generation using DL. While most studies achieved good scores in technical aspects such as model design and evaluation metrics, they often lacked reproducibility components like open-source code and external validation. This limits the generalizability and clinical credibility of the proposed approaches. By systematically assessing these aspects, our review not only synthesizes recent advances but also exposes areas that require improvement for future translational research. The availability of a standardized evaluation like METRICS can help raise methodological standards and guide the design of more robust and clinically meaningful AI algorithms.

The PET images generated via advanced DL architectures represent a major leap forward in medical imaging, providing a variety of methods to achieve high-quality synthetic images. As shown in Table 4, various studies have investigated DL architectures for synthetic PET generation, leveraging different input modalities and tracer types across brain and whole-body applications. Figure 2 illustrates the distinct trends between these two domains.

**Table 4:** Summary of studies on synthetic PET image generation (2019–2024)

Input modality	Output modality	Deep learning architecture	Clinical focus/district	Authors [reference number]
Low-dose PET - CT	Full-dose PET	2D U-Net	Whole body	Haggstrom et al [43]
Low-dose PET - MRI	Full-dose PET	2D cGAN	Whole body	Kaplan and Zhu [4]
Low-dose PET - MRI	Full-dose PET	3D cGAN	Brain	Wang et al [47]
Low-dose PET - MRI	Full-dose PET	3D CNN	Brain	Chen et al [34]
MRI	11C-PIB PET	3D cGAN	Brain	Wei et al [48]
Low-dose PET - CT	Full-dose PET	3D U-Net	Brain	Sanaat et al [49]
MRI	Full-dose PET	2D cGAN	Brain	Shin et al [50]
MRI	Full-dose PET	2D U-Net	Brain	Emami et al [44]
Low-dose PET	Full-dose PET	GAN	Brain	Islam and Zhang [51]
Low-dose PET - CT	Full-dose PET	2D CycleGAN	Whole body	Xue et al [24]
Low-dose PET - MRI	Full-dose PET	3D CNN	Whole body	Wang et al [38]
MRI	Full-dose PET	2D ViT	Brain	Zhang et al [52]
MRI	Full-dose PET	3D cGAN	Brain	Sikka et al [11]
MRI	Full-dose PET	3D cGAN	Brain	Hu et al [12]
Low-dose PET - MRI	Full-dose PET	3D cGAN	Brain	Lin et al [53]
Full-dose PET - MRI	11C-UCB-J PET	3D U-Net	Brain	Wang et al [42]
Low-dose PET	Full-dose PET	3D cGAN	Whole body	Zhou et al [36]
Low-dose PET - MRI	Full-dose PET	2D cGAN	Brain	Sun et al [54]
Low-dose PET	Full-dose PET	3D GAN	Whole body	Abazari et al [37]
MRI	Full-dose PET	3D BPGAN	Brain	Zhang et al [15]
MRI	Tau PET	2D LDM	Brain	Jang et al [33]
MRI	Full-dose PET	2D DDPM	Brain	Xie et al [16]
MRI	Full-dose PET	3D U-Net	Whole body	Rajagopal et al [20]
Full-dose PET	11C-UCB-J, 11C-PiB PET	3D CNN	Brain	Zhang et al [3]
Low-dose PET	Full-dose PET	3D U-Net	Brain	Kim et al [23]
Low-dose PET - MRI	Full-dose PET	3D MLG-GAN	Brain	Gao et al [55]
Static PET	Dynamic PET	3D cycleGAN	Whole body	Miao et al [35]
MRI	Full-dose PET	2D EA-GAN	Whole body	Lei et al [25]
MRI	Methionine PET	3D GAN	Brain	Takita et al [56]
Full-dose PET - MRI	Tau PET	3D CNN	Brain	Lee et al [40]
MRI	Full-dose PET	2D U-Net	Brain	Wang et al [57]
MRI	Full-dose PET	2D cGAN	Brain	Vega et al [22]
MRI	15O-water PET CBF	3D CNN	Brain	Hussein et al [19]
MRI	Full-dose PET	3D CNN	Brain	Ouyang et al [58]

The table highlights various input-output combinations used in synthetic PET generation, covering a range of deep learning architectures. The studies are categorized based on the clinical focus/district involved and the imaging modalities employed

*CT* Computed tomography, *cGAN* Conditional generative adversarial network, *CNN* Convolutional neural network, *DDPM* Denoising diffusion probabilistic model, EA-GAN Edge-aware generative adversarial network, *GAN* Generative adversarial network, *LDM* Latent diffusion model, *MRI* Magnetic resonance imaging, *MLG-GAN* Multi-level generative adversarial network, *PiB* Pittsburgh compound B, *PET* Positron emission tomography, *ViT* Vision transformer

The majority of studies focus on brain imaging, where low-dose PET, MRI, and CT are commonly used as input modalities to generate full-dose PET or specialized PET tracers, such as tau PET [33], 11C-UCB-J PET [34], or dynamic PET scans [35]. This emphasis reflects the growing demand for advanced neuroimaging techniques to enhance diagnosis and disease monitoring in neurological disorders. Additionally, the widespread adoption of synthetic PET in brain imaging highlights its potential to improve diagnostic accuracy while minimizing radiation exposure, a particularly relevant factor in neurology, where high-resolution imaging is essential for understanding disease progression. Although whole-body synthetic PET applications are less prevalent, they remain a key area of investigation, particularly in oncology [36, 37]. The ability to synthesize high-quality PET images for oncological assessments suggests potential clinical benefits, such as enhanced tumor detection and reduced radiation exposure. As DL methods continue to evolve, future research is expected to expand whole-body synthetic PET applications, improving diagnostic workflows and broadening clinical adoption.

The architectures used indicate that 3D cGANs are frequently applied for whole-body imaging, translating low-dose PET images into full-dose PET images. These models capture detailed spatial relationships in PET data while retaining clinically relevant information, as demonstrated in studies by Zhou et al (2022) and Wang et al [36, 38]. The integration of MRI data as input, in addition to PET images, offers enhanced anatomical detail for improved visualization in synthetic PET [15]. The versatility of 3D cGANs has been shown in malignancy characterization using whole-body MR images [5].

In contrast, 2D cGANs and U-Net architectures are more commonly utilized for brain imaging, where input images are often MRI or CT scans. These architectures demonstrate adaptability to the specific anatomical requirements of brain PET synthesis, emphasizing their suitability for capturing finer details. The capacity of GANs to generate diverse, realistic samples makes them particularly valuable in medical fields where large public datasets are scarce. For instance, the “globally and locally aware” GAN model incorporates global and local modules to enhance synthesis quality by addressing anatomical details on multiple scales, as reported by Sikka et al in 2021 [11].

Only a limited number of studies explored multi-tracer PET synthesis (*e.g*., tau, amyloid, synaptic density tracers), despite their growing importance in neurodegenerative disease imaging. Likewise, advanced architectures such as vision transformers and diffusion models, although promising, remain underutilized, with limited evaluation of robustness and generalizability. Most included studies were centered on neuroimaging, reflecting an imbalance in clinical application domains. These observations suggest opportunities for future work in underrepresented areas such as whole-body imaging and tracer-specific synthesis beyond FDG.

The synthesis methods also vary in the tracers used. Many studies focus on synthesizing images for ^18^F-FDG PET, essential for diagnosing metabolic conditions, especially in neurological disorders [11]. Other studies have explored tracers such as 11C-UCB-J for assessing synaptic density and astrocytosis or gliosis [38] and ^11^C-PIB for amyloid deposit detection [20]. Another study focuses on ^15^O-H_2_O PET, used to measure cerebral blood flow, which is fundamental in brain function analysis [39]. Tau tracers, as employed in studies by Lee et al and Jang et al [33, 40], play a role in distinguishing patients with rapidly progressive dementia due to Alzheimer’s disease from those with lower neurofibrillary tangle burdens [41]. This growing interest in using synthetic PET to study specific pathologies like Alzheimer’s disease and other neurodegenerative disorders suggests the importance of these biomarkers for early diagnosis and disease monitoring.

The alluvial plot in Fig. 3 reveals that MRI is the predominant input modality for generating various types of synthetic PET images. For this reason, the pie chart in Fig. 4 illustrates the distribution of MRI modalities used in synthetic PET studies, with T1-weighted images dominating at 63.6%. This preference highlights the extensive utility of T1-weighted imaging in PET synthesis tasks due to its superior structural resolution and compatibility with DL models. For instance, studies like Vega et al (2024) demonstrate that synthetic full-dose PET images generated from T1-weighted brain MRI can improve the performance of deep anomaly detection models in neuroimaging applications [22].

Diffusion-weighted imaging (DWI) accounts for 9.1% of inputs, reflecting its importance in capturing microstructural details that enhance PET synthesis for specific applications, such as neurological diagnostics.

Modalities like Magnetization Transfer Ratio (MTR) represent 3%, reflecting their niche role in addressing specialized imaging needs, particularly in the context of tissue characterization. Meanwhile, Arterial Spin Labeling (ASL), T2-weighted, T2-FLAIR, and T1 post-contrast imaging each comprise 6.1% of the inputs, illustrating their focused application in scenarios requiring enhanced contrast or functional information.

All metrics play a key role in evaluating network performance, but our analysis focuses on the four most common metrics—PSNR, SSIM, MAE, and rRMSE—which are widely recognized in image synthesis literature.

A 3D U-Net architecture [42] achieved the highest performance in PSNR, demonstrating the effectiveness of U-Net-based models for preserving spatial features in synthetic PET images. Similarly, Haggstrom et al [43] used a 2D U-Net and showed noteworthy performance, underscoring the model’s robustness in low-dose to full-dose PET synthesis.

CycleGAN architectures, like the 2D CycleGAN used by Xue et al [24], excel in SSIM, showing a strong ability to retain high-level and fine-grained details.

A 3D cGAN architecture [38], when combined with MRI input for full-dose PET synthesis, performed best in minimizing error, as reflected by its low MAE values. In terms of rRMSE, a 3D CNN architecture [15] outperformed others in brain-region PET image synthesis, demonstrating its ability to maintain high numerical accuracy, essential in diagnosing neurological conditions that require precise spatial representation.

U-Net architectures consistently perform well across multiple studies, confirming their efficiency in preserving spatial hierarchies within image data. Emami et al [44] introduced a frequency-aware U-Net (FREA-UNet), which optimizes synthesis by managing both low- and high-frequency components, resulting in improved image quality and detail preservation. New Transformer-based models, as explored by Li et al (2023) and Shamshad et al (2022), show potential in enhancing feature extraction through spatial attention mechanisms [45, 46].

A comparative analysis of the reviewed studies suggests that U-Net-based architectures, both 2D and 3D, consistently deliver strong results in terms of PSNR and SSIM, particularly for low-dose PET to full-dose PET synthesis in both brain and whole-body applications. Their encoder-decoder structure with skip connections supports the preservation of spatial resolution and anatomical detail. GAN-based models (especially cGANs and CycleGANs) tend to excel in preserving texture and producing visually realistic outputs, often achieving superior SSIM values in MRI-to-PET translation, although they may exhibit instability during training and lack precise control over intensity values. Transformer-based models and Diffusion models, although less common in the current literature, demonstrate promising results in handling complex multimodal inputs and capturing global contextual features. However, they require substantial computational resources and are still under-evaluated in terms of robustness and clinical applicability. These observations underscore that model selection should be guided not only by architecture but also by the clinical context and data availability.

The clinical deployment of AI-based synthetic PET models raises important regulatory, ethical, and interpretability concerns. As these systems may influence diagnostic decisions, questions of responsibility, transparency, and patient safety become critical. Most reviewed studies do not address model explainability or mechanisms for interpreting output decisions, which limits their acceptability in clinical workflows. Furthermore, regulatory frameworks such as the FDA’s guidance on AI/ML-based software as a medical device (SaMD) and the upcoming EU AI Act require rigorous validation, performance monitoring, and risk management before clinical adoption. Meeting these requirements will necessitate greater attention to documentation, human oversight, and post-deployment auditing. Future work should incorporate not only technical performance but also compliance pathways and strategies to ensure AI systems are ethically and legally deployable in real-world settings.

However, further evaluation is required to compare their performance in terms of PSNR and SSIM against well-established architectures like U-Net and GAN-based models. Additionally, diffusion models are emerging as novel approaches for PET image generation, though results are still limited in the current literature.

This review highlights promising advancements in synthetic PET imaging using DL, with several studies demonstrating the potential for high-quality image generation and substantially reduced radiation exposure. These developments are particularly significant in pediatric populations, where minimizing radiation dose is crucial to ensure patient safety and long-term health. Nonetheless, methodological variability and limited clinical validation continue to pose substantial challenges. Future research should prioritize the development of standardized evaluation protocols, the use of larger and more diverse datasets—including pediatric cohorts—and comprehensive real-world clinical validation to support the safe and effective translation of synthetic PET techniques into clinical practice.

## Abbreviations

cGAN: Conditional generative adversarial network
CNN: Convolutional neural network
CT: Computed tomography
FDG: Fluorodeoxyglucose
GAN: Generative adversarial network
MAE: Mean absolute error
MRI: Magnetic resonance imaging
PET: Positron emission tomography
PSNR: Peak signal-to-noise ratio
rRMSE: Relative root mean square error
SSIM: Structural similarity index
SUV: Standardized uptake value
